# Nature’s Microfabricated 3D Coupled Dual-Membrane Resonators—A Dynamic Analysis of Circular Diatom Frustules

**DOI:** 10.3390/mi17101134

**Published:** 2026-09-29

**Authors:** Yu Jia

**Affiliations:** School of Engineering and Innovation, Aston University, Birmingham B4 7ET, UK; y.jia1@aston.ac.uk

**Keywords:** diatom frustules, resonator, 3D MEMS

## Abstract

MEMS (microelectromechanical system) resonators, meticulously fabricated by advanced lithographic and etching processes, have become indispensable technological cornerstones across modern sensing, time-keeping and actuation applications. Yet, realising 3D coupled architectures for these silicon-based resonators remains a fundamental microfabrication challenge, limiting the toolkit of exploitable dynamic phenomena to the in-plane and flexural modes. Nature, however, through 200 million years of evolutionary refinement, has nano-engineered a ready-made solution: the diatom frustule made from biosilica (SiO_2_), a robust material with mechanical properties well-suited to micro-mechanical resonator applications. Unlike planar MEMS counterparts, frustules are typically dual-membrane structures, vertically coupled by a girdle band, introducing complex 3D coupling dynamics that are prohibitively complex to achieve with MEMS fabrication. This paper explores the analytical and numerical modelling of a typical circular frustule’s dynamic behaviour. Alongside the typical transverse modes expected from MEMS resonators, the 3D coupled structure also exhibits unique girdle band tilt modes, exclusively activated by antisymmetric excitations. Collectively, the presence of partial mode localisation, mechanical energy amplification through the low-inertia girdle band, and rotational–transverse modal coupling, demonstrates the diatom frustule to be a dynamically rich resonator. This establishes the theoretical framework for exploiting the diatom frustule as a novel bio-inspired and bio-derived 3D resonator building block.

## 1. Introduction

Microscopic mechanical resonators fabricated using microelectromechanical system (MEMS) processes have become a key cornerstone of modern technology in the past few decades [1]. These intricately micro-engineered vibrating mechanisms underpin a wide range of technologies including: inertial sensors [2], accelerometers, gyroscopes, magnetometers, pressure sensors [3], microphones, environmental sensors, optical sensors, micro-mirrors, biosensors [4], microbalances, ultrasonic actuators, energy harvesters [5], time-keeping [6], and many more [7]. Fuelled by advancements in microfabrication, such as the landmark DRIE breakthrough in the 1990s [8], MEMS witnessed a rapid uptake across multiple sectors such as automotive, aerospace, consumer electronics, defence, industrial, healthcare, telecommunication, etc., with a global market size projected to attain 22 billion USD by 2030 [7].

Nonetheless, achieving microfabrication is by no means a straightforward achievement. From photolithography to etching, deposition to packaging, cleanroom facilities to characterisation labs, every single process step is a substantial and meticulous undertaking in terms of both engineering complexity and operational cost. Despite this, the dominant wisdom within MEMS design today is primarily based on 2D topology due to the complexities involved with fabricating complex 3D mechanisms.

Vertically stacked 3D microsystems can get prohibitively complex and costly to fabricate, while also holding immense untapped potentials for next-generation devices [9]. For example, submicron reconfigurable structures [10] and tunable 3D architectures [11] have been demonstrated to offer new avenues of performance. 3D-printed techniques offer fabrication resolution down to tens of microns, while the combination of 3D printing and other micromachining techniques, such as magnetron sputtering [12], has shown relatively more precise and rapid prototyping capabilities. 3D printing using two-photo polymerisation techniques has also been demonstrated for NEMS devices. Alternatively, 2D planar structures can be assembled post-fabrication to form 3D structures, with recent advances in novel 3D assembly techniques that leverage predefined loading conditions [13] and multi-level strategies [14]. These advances in 3D printing and 3D assembly illustrate a need for 3D architectures to unlock better performance and additional functionalities, despite the need to overcome fabrication hurdles.

**Figure 1:**
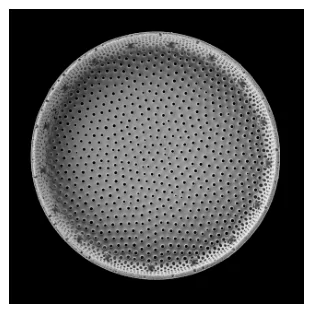
Scanning electron micrograph of a frustule valve face, showing the characteristic hexagonal areolae pore array. (Image credit: Pavel Somov, Wikimedia Commons, CC BY 4.0, accessed on 9 September 2026: https://tinyurl.com/m9sf6cb6) Estimated diameter of 40 μm.

In the meantime, nature, operating through hundreds of millions of years of evolutionary optimisation, has arrived at complex micro/nano structural solutions that rival, and in some cases, surpass the state-of-the-art engineered microsystems. One such example is the diatom frustule, evolved over 200 million years ago [15]. Diatoms are unicellular eukaryotic microalgae organisms [16], which form a hard and porous external wall structure encasing its organic matter. This shell around diatoms is called a frustule, composed almost entirely of silica. An example of one-half of the frustule valve is shown in Figure 1, illustrating a strikingly organised structure. A wide variety of well-defined shapes exist amongst diatoms, including the circular disc shape shown in this image.

The diatom frustule is made of biosilica, also known as biogenic silica. It is an amorphous silica shell created by diatoms while alive. When its organic matter is flushed and dehydrated, the frustule is predominantly composed of SiO_2_. There might be residual nanoscopic traces of water H_2_O and organic matrix associated with silanol groups Si-OH. Overall, the mechanical property of frustules can be approximated to typical amorphous silica.

**Figure 2:**
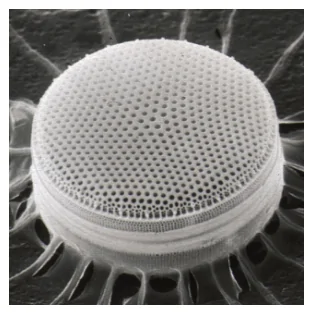
Scanning electron micrograph of a circular diatom frustule. (Image credit: CSIRO, Wikimedia Commons Attribution 3.0 Unported, accessed on 9 September 2026: https://tinyurl.com/yudm3unn) Estimated diameter of 40 μm.

A full diatom frustule is made up of two similarly shaped valves called theca, joined together by girdle bands as thin vertical walls. After diatoms die, the organic material can be flushed and the diatom frustule becomes a purely mechanical biosilica structure as shown in Figure 2. During the silicification process, frustules can form one of a vast range of shapes, including circles, triangles, squares, spindles, ellipses and many more. They will almost always have an array of neatly patterned pores on both thecae [17].

One can consider the microscopically sized diatom frustule as somewhat analogous to the macroscopically sized seashells. Both are hard outer structures constructed by their organic internal residents. However, seashells are mainly made up of calcium carbonate (CaCO_3_). By comparison, silica is a much harder and stiffer mechanical material. The porous structure also makes the frustule a less dense material. Most importantly, silica has substantially higher quality factor compared to calcium carbonate [18], while a seashell’s calcium carbonate has high internal damping from the layered organic matrix [19]. Therefore, biosilica’s mechanical performance, being predominantly composed of SiO_2_, has the potential to be comparable to annealed fused silica or MEMS-grade silicon.

Finite element modelling [20] and experimental probing [21] have been studied to estimate the resonance frequencies and mode shapes of diatom frustules. These studies are confined to eigenfrequency and modal analysis, without studying diatom frustule’s 3D dynamics. The fundamental resonant modes are estimated to range between 1 MHz and 8 MHz. Finite element modelling of frustule [22] illustrated rich vibratory dynamics and resonant mode shapes, which has already led to biologically inspired microsystem designs [23]. Characterisation of mechanical properties using AFM and SEM [24] show a heterogeneous spread in elastic modulus, stiffness and density, depending on porosity, hydration and organic content.

Experimental cyclic loading [24] of cleaned frustule demonstrated an oscillatory response, showing potential promise as a micro/nano-resonator. A hybrid XCT and SEM experimental characterisation method determined the Young’s modulus and observed a clear elastic deformation region [25]. However, more experimental investigation is still required to determine its robustness and long-term vibratory stability.

This paper, for the first time, establishes the analytical model and explores the numerically simulated dynamic behaviour of the dual-membrane coupled resonator structure found in diatom frustules. This will help to determine the vibratory suitability of such a 3D structure as a fundamental building block for biogenic micro-mechanical resonators.

A key criticism of employing such biogenic structures could be the geometric diversity and heterogeneity amongst each individual frustule. To be exploited as a resonator, each device will require individual calibration and tuning. However, such a drawback exists with MEMS resonators too due to fabrication tolerance and location on the wafer, albeit at a much smaller level of non-uniformity. Nonetheless, where precision and stable metrology is required, each individual MEMS resonator still needs to be periodically calibrated.

## 2. Analytical Model

**Figure 3:**
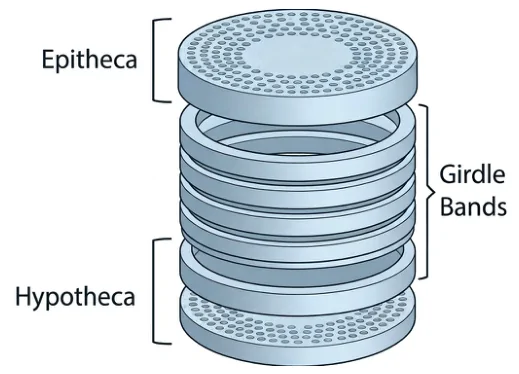
Exploded view of a generic model of circular diatom frustules.

For a given circular diatom frustule, there are two overlapping valves known as thecae. The upper valve is the epitheca; the bottom valve is the hypotheca. The two valves are vertically and circumferentially connected by girdle bands. This can be analytically represented by two circular disc plates that are vertically coupled by girdle bands. Figure 3 illustrates an exploded view of a generic model representation for this dual-membrane structure, based off similar shapes explored in the literature [22].

### 2.1. Circular Membrane Resonator

The Kirchhoff–Love plate theory can be used as the starting point to analytically model the dynamic behaviour of each circular valve (membrane disc plate). Each valve can be reduced to Equation (1) in polar coordinates.

(1) $$D\nabla^{4}w\left(r,\theta ,t\right)+\rho h\frac{\partial^{2}w}{\partial t^{2}}+c\frac{\partial w}{\partial t}=p\left(r,\theta ,t\right)$$

where w(r,θ,t) is transverse displacement, *r* is radial coordinate with 0≤r≤R, *R* is radius, θ is angular coordinate with 0≤θ<2π, *t* is time, *D* is effective flexural rigidity defined by Equation (2), ρ is effective mass density of the porous valve, *h* is valve (plate) thickness, *c* is viscous damping, and *p* is externally applied transverse pressure (p=0 for free vibration analysis).

(2) $$D=\frac{Eh^{3}}{12(1-\nu^{2})}$$

where ν is the Poisson’s ratio, *E* is the effective Young’s modulus. Diatom frustules are made of SiO_2_. Therefore, both *E* and ρ values should be close to typical silica values. Furthermore, frustules should be flushed of organic matter and dehydrated before being used as a mechanical resonator, to minimise any non-silica contaminants. However, any residual nanoscopic liquid and organic matter could slightly alter the effective density and elastic moduli values, which can be represented by Equations (3) and (4) using the non-homogenisation factor φ.

(3) $$E=E_{SiO_{2}}\left(1-\varphi \right)$$

(4) $$\rho =\rho_{SiO_{2}}\left(1-\varphi \right)$$

From Equation (1), $\nabla^{4}$ is the fourth-order partial differential equation known as the biharmonic operator or the bilaplacian operator, derived from applying the scalar Laplacian $\nabla^{2}$ twice. These are expanded in Equations (5) and (6). The fourth-order spatial characterisation thus enables the two-dimensional analytical modelling of the plates, requiring two boundary conditions per edge.

(5) $$\nabla^{2}=\frac{\partial^{2}}{\partial r^{2}}+\frac{\partial }{r\partial r}+\frac{\partial^{2}}{r^{2}\partial \theta^{2}}$$

(6) $$\nabla^{4}w=\frac{\partial^{4}w}{\partial r^{4}}+\frac{2\partial^{3}w}{r\partial r^{3}}-\frac{\partial^{2}w}{r^{2}\partial r^{2}}+\frac{\partial w}{r^{3}\partial r}+\frac{2\partial^{4}w}{r^{2}\partial r^{2}\partial \theta^{2}}-\frac{2\partial^{3}w}{r^{3}\partial r\partial \theta^{2}}+\frac{4\partial^{2}w}{r^{4}\partial \theta^{2}}+\frac{\partial^{4}w}{r^{4}\partial \theta^{4}}$$

For axisymmetric modes with no angular dependence ($\frac{\partial }{\partial \theta }\equiv 0$), all θ-derivative terms vanish and the PDE can be simplified to Equation (7).

(7) $$D(\frac{{\;d}^{4}w}{\;dr^{4}}+\frac{2}{r}\frac{{\;d}^{3}w}{\;dr^{3}}-\frac{1}{r^{2}}\frac{{\;d}^{2}w}{\;dr^{2}}+\frac{1}{r^{3}}\frac{\;dw}{\;dr})+\rho h\frac{{\;d}^{2}w}{\;dt^{2}}+c\frac{\;dw}{\;dt}=p(r,t)$$

### 2.2. Dual Membranes Vertically Coupled by Girdle Band

The coupling of the top and bottom membrane plates by the girdle band can be represented by four ODEs as shown in Equations (8)–(11) following the Galerkin method to reduce the distributed model to a single generalised coordinate $q_{i}\left(t\right)$. Displacements $q_{1}$ and $q_{2}$ represent the top plate and bottom plate respectively, while the interconnecting girdle band has displacements $q_{g}$ in the axial direction and $\theta_{g}$ in the angular tilt direction.

(8) $$m_{1}\ddot{q_{1}}+c_{v}\dot{q_{1}}+\left(k_{1}+k_{z}\right)q_{1}-k_{z}q_{g}+k_{\phi }\theta_{g}=F_{1}\left(t\right)$$

(9) $$m_{2}\ddot{q_{2}}+c_{v}\dot{q_{2}}+\left(k_{2}+k_{z}\right)q_{2}-k_{z}q_{g}-k_{\phi }\theta_{g}=F_{2}\left(t\right)$$

(10) $$m_{g}{\ddot{q}}_{g}+c_{g}{\dot{q}}_{g}+2k_{z}q_{g}-k_{z}q_{1}-k_{z}q_{2}=0$$

(11) $$I_{g}{\ddot{\theta }}_{g}+c_{\phi }{\dot{\theta }}_{g}+2k_{\phi }\theta_{g}+k_{\phi }q_{1}-k_{\phi }q_{2}=0$$

where $m_{i}$ is the generalised modal mass (not physical mass) of the fundamental modal shape Φ(r) of the circular valve (i=1 for upper valve, and i=2 for lower valve) given by Equation (12); $c_{v}$ is the valve viscous damping; $c_{g}$ is the girdle band axial damping; $c_{\phi }$ is the girdle band rotational damping; $k_{i}$ is the generalised modal stiffness of a single valve in isolation given by Equation (13); $k_{z}$ is the girdle band axial direction coupling stiffness given by Equation (14); $k_{\phi }$ is the girdle band rotational coupling stiffness given by Equation (15); $m_{g}$ is the girdle band mass given by Equation (16); $I_{g}$ is the girdle band rotational moment of inertia given by Equation (17); and $F_{i}$ is the generalised modal force given by Equation (18).

(12) $$m_{i}=\rho h\int_{0}^{R}\Phi^{2}\left(r\right)2\pi rdx$$

(13) $$k_{i}=D\int_{0}^{R}{\left(\nabla^{2}\Phi \right)}^{2}2\pi rdx$$

(14) $$k_{z}=\frac{2\pi RE_{g}t_{g}}{H}$$

where $E_{g}$ is Young’s modulus of the girdle band, $t_{g}$ is the girdle band wall thickness, and *H* is the girdle band height.

(15) $$k_{\phi }=\frac{\pi RE_{g}t_{g}^{3}}{6H}$$

(16) $$m_{g}=s\phi RHt_{g}\rho$$

(17) $$I_{g}=\frac{m_{g}R^{2}}{2}$$

(18) $$F_{i}=\int_{0}^{R}p_{i}\left(r,t\right)\Phi \left(r\right)2\pi rdr$$

where $p_{i}$ is the actual pressure (or force per unit area) acting on each of the valves.

### 2.3. Modal Coupling

At the core of coupled oscillatory systems, it is key to explore the behaviour phenomena when $k_{1}$ and $k_{2}$ approach each other. As a starting point, the governing equations can first be reduced to a 2 DOF valve-only system by treating the girdle band as a massless coupling spring (valid when $m_{g}<<m$), as shown in Equations (19) and (20).

(19) $$m_{1}{\ddot{q}}_{1}+k_{1}q_{1}-k_{c}q_{2}=0$$

(20) $$m_{2}{\ddot{q}}_{2}+k_{2}q_{2}-k_{c}q_{1}=0$$

where $k_{c}$ is the effective inter-valve coupling stiffness defined by Equation (21), and captures the combined axial and rotational transmission of the girdle band.

(21) $$k_{c}=k_{z}+\frac{k_{\phi }}{R^{2}}$$

The exact eigenfrequency solution is given by Equation (22), governing various dynamic phenomena such as modal localisation, modal coupling and mode decomposition.

(22) $$\omega_{\pm }^{2}=\frac{k_{1}+k_{2}}{2m}\pm \frac{1}{2m}\sqrt{{\left(k_{1}-k_{2}\right)}^{2}+4k_{c}^{2}}$$

#### 2.3.1. Modal Localisation

Modal localisation is the tendency of vibratory energy in a coupled resonator system to concentrate on one oscillator rather than being shared equally between them. In this context, modal localisation concentrates energy spatially in one valve rather than being shared between both. The localisation parameter $L_{\pm }$ can be described by Equations (23) and (24).

(23) $$L_{+}=\frac{\delta k}{\sqrt{\delta k^{2}+4k_{c}^{2}}}$$

(24) $$L_{-}=\frac{-\delta k}{\sqrt{\delta k^{2}+4k_{c}^{2}}}$$

where δk is the stiffness mismatch ($k_{1}-k_{2}$) between the two valves.

Three regimes can be observed:

- When there is strong coupling, $k_{c}>>\delta k$, $L_{+}\approx 0$; energy is equally shared and modes are delocalised.
- During transition, $k_{c}=\delta k$, $L_{+}=\frac{1}{\sqrt{2}}$, half localised.
- Weak coupling, $k_{c}<<\delta k$, $L_{+}\approx 1$, fully localised to valve 1.

#### 2.3.2. Modal Coupling and the Avoided Crossing

As δk−>0, the two frequencies approach but never cross as they repel each other. The minimum separation is set by $k_{c}$. The frequency splitting Δω at any given mismatch δk is given by Equation (25).

(25) $$\Delta \omega =\omega_{+}-\omega_{-}\approx \frac{\delta k^{2}+4k_{c}^{2}}{2m\sqrt{\frac{(k_{1}+k_{2})/2}{m}}}$$

At exact degeneracy δk=0, Δω becomes as shown in Equation (26).

(26) $${\Delta \omega |}_{\delta k=0}=\frac{2k_{c}}{2m\sqrt{\frac{(k_{1}+k_{2})/2}{m}}}$$

The avoided crossing curve as a function of δk is a hyperbola given in Equation (27).

(27) $$\Delta \omega^{2}=(\frac{\delta k}{2m\sqrt{\frac{(k_{1}+k_{2})/2}{m}}}{)}^{2}+(\frac{k_{c}}{m\sqrt{\frac{(k_{1}+k_{2})/2}{m}}}{)}^{2}$$

The asymptotes of this hyperbola (δk−>±inf) correspond to the two uncoupled valve frequencies. The gap at δk=0 is entirely determined by $k_{c}$, the girdle band coupling stiffness. This is difficult to physically manifest in conventional MEMS structures due to fabrication complexities. Therefore, this 3D coupling has the potential to open new avenues of dynamic behaviour for coupled resonators.

#### 2.3.3. Mode Decomposition and Mixing Angle

Mode decomposition expresses the physical valve displacements ($q_{1}$, $q_{2}$) as projections onto the system’s normal mode coordinates ($p_{+},p_{-}$) via rotation transformation whose mixing angle α (Equation (28)) quantifies the degree to which the true eigenmodes of the asymmetric coupled system deviate from the idealised symmetric and antisymmetric basis, transitioning continuously from $\alpha =45^{\circ }$ (identical valves, equal energy sharing) to $\alpha =0^{\circ }$ (fully mismatched valves, complete mode localisation).

(28) $$\tan2\alpha =\frac{2k_{c}}{\delta k}$$

Consider three various cases:

- Identical valves: δk=0; $\alpha =45^{\circ }$; pure antisymmetric and symmetric modes; share energy equally.
- Large mismatch, strong coupling: $\delta k<<k_{c}$; $\alpha \approx 45^{\circ }-\frac{\delta k}{4k_{c}}$; modes are nearly symmetric/antisymmetric; slightly rotated.
- Large mismatch, weak coupling: $\delta k>>k_{c}$; $\alpha \approx \frac{k_{c}}{\delta k}\approx 0$; each mode is fully localised to one valve and coupling $k_{c}$ is essentially irrelevant.

## 3. Numerical Models

The scope of the model explored here is focused on the transverse modes of valve 1 and valve 2: girdle axial mode and girdle tilt mode. This explores the coupling between inter-valve coupling, axial girdle coupling and rotational–transverse coupling.

The material properties and parameters in Table 1 were used for numerical modelling. Material properties were taken from the COMSOL Multiphysics 6.2 library for silica glass, as a close approximation to biosilica. Quality factors were assumed values for reasonable resonators.

### 3.1. Finite Element Analysis of Mode Shapes

**Figure 4:**
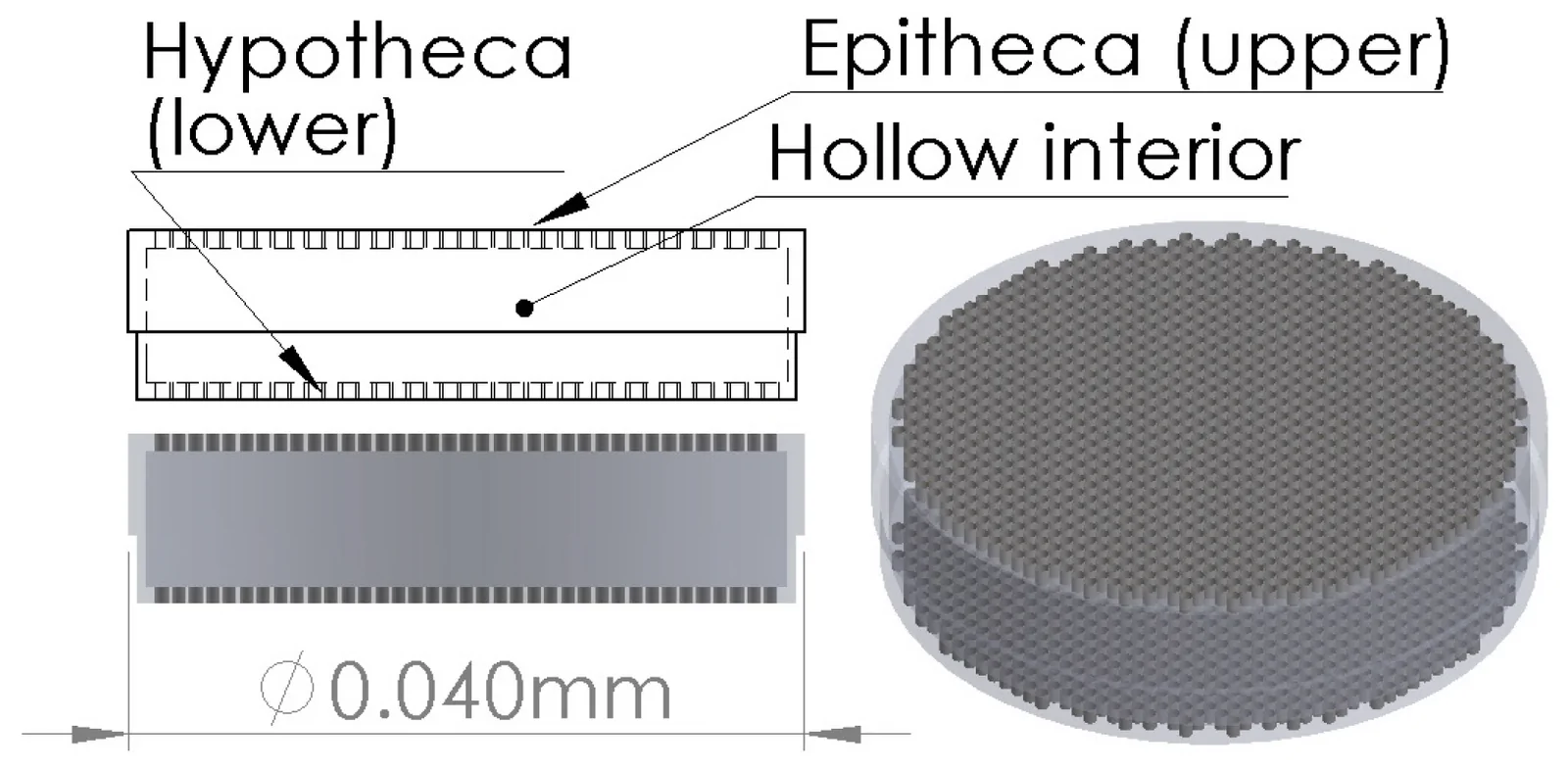
CAD model of a circular diatom frustule.

A CAD model (Figure 4) was drawn based on a generic model representation of a typical circular diatom frustule. Both the top valve (epitheca) and the bottom valve (hypotheca) are perforated with an array of holes typical of the pores found in such structures. Radii of both thecae were estimated from SEM images, with epitheca adopting 20 μm and hypotheca taking 19.5 μm. Valve thickness was set at 1 μm.

A finite element model of this structure was constructed in COMSOL, using typical SiO_2_ material properties. The circumferential centre edge line of the girdle band was set as the fixed constraint, allowing the valves and the band walls to vibrate. This was chosen as the fixed constraint to allow both top and bottom plates to vibrate freely, while also allowing most of the vertical wall the freedom to vibrate. Eigenfrequency analysis of up to the first 100 modes was carried out with an extremely fine mesh that covered the smallest geometric features of this intricate structure.

**Figure 5:**
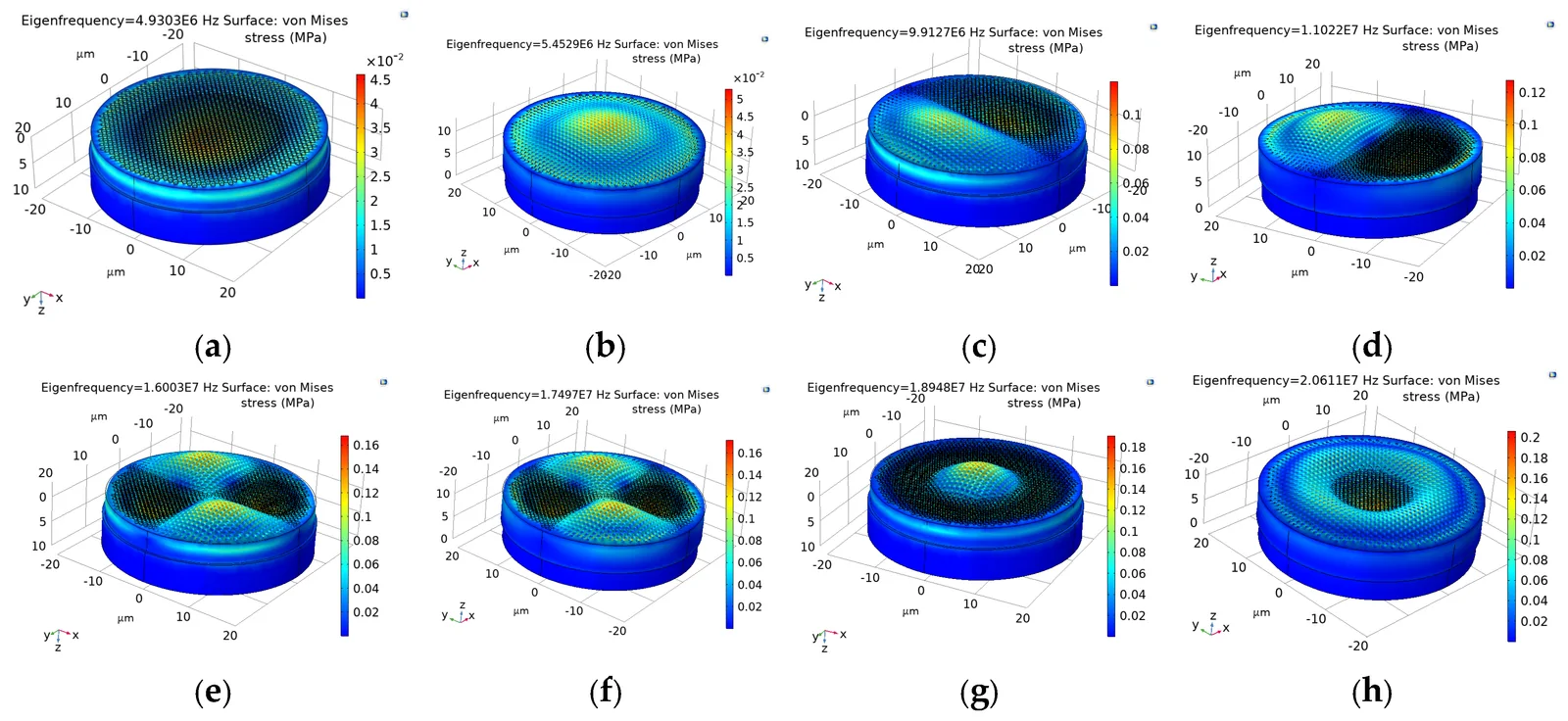
Finite element analysis of the first 4 resonant mode shapes of the 3D coupled resonators, illustrating typical disc membrane behaviour. The standard mode shape naming convention (n,m) for circular discs is employed here, where *n* denotes number of nodal lines and *m* denotes number of nodal circles. (**a**) Bottom valve mode (0, 1), 4.9 MHz. (**b**) Top valve mode (0, 1), 5.5 MHz. (**c**) Bottom valve mode (1, 1), 9.9 MHz. (**d**) Top valve mode (1, 1), 11.0 MHz. (**e**) Bottom valve mode (2, 1), 16.0 MHz. (**f**) Top valve mode (2, 1), 17.5 MHz. (**g**) Bottom valve mode (0, 2), 18.9 MHz. (**h**) Top valve mode (0, 2), 20.6 MHz.

Figure 5 shows the first four resonant modes of the overall structure, showing good agreement with circular membrane modes as per classical plate theory. Modes (1, 1) and (2, 1) also have nearby decoupled twin modes, with mode shapes shifted by 90° due to geometric asymmetry from the mesh. In practice, this geometric asymmetry would be even more pronounced. In MEMS counterparts, there is almost certainly geometric asymmetry and similar modal decoupling as well due to fabrication tolerances.

**Figure 6:**
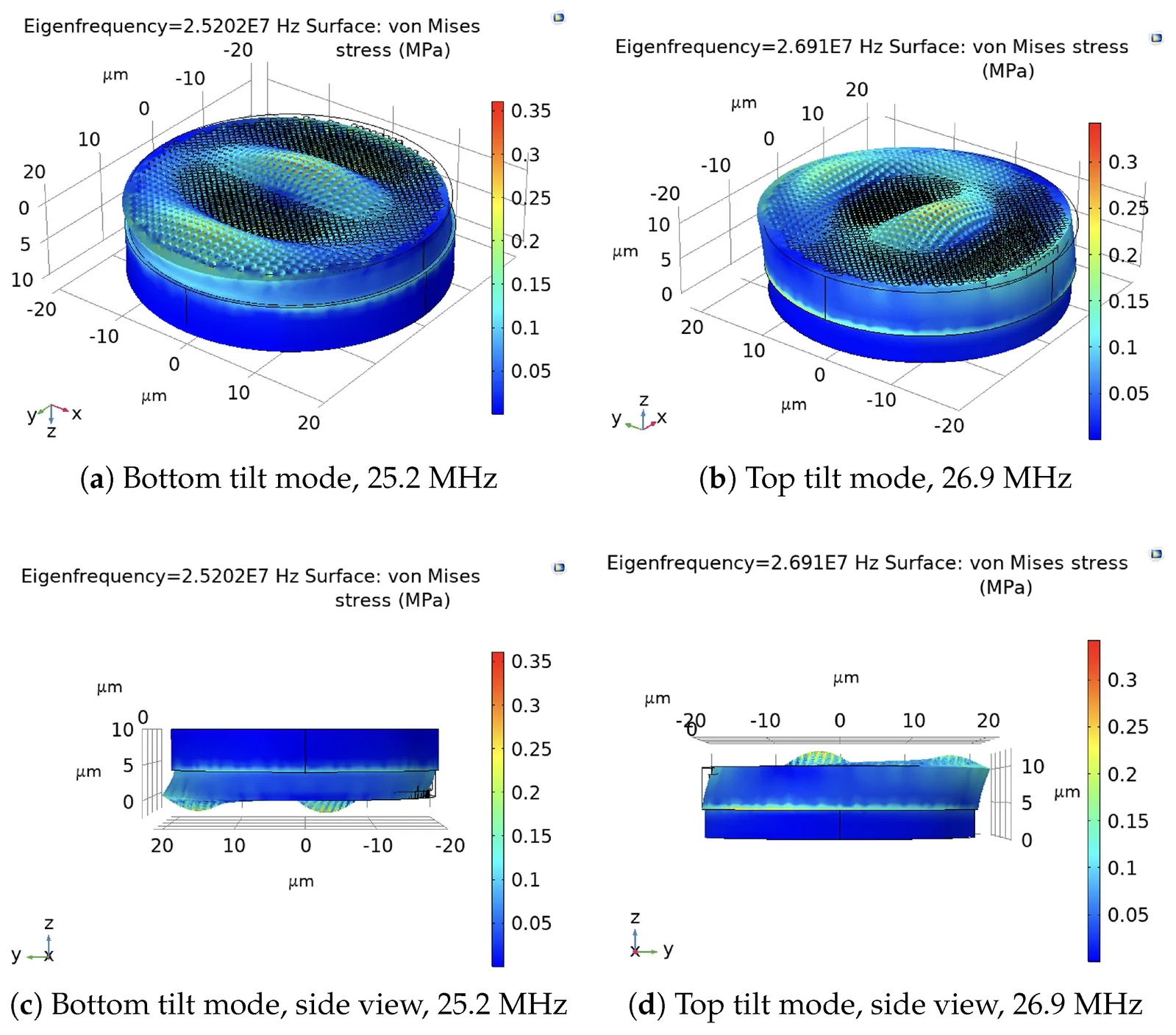
Finite element analysis of tilt modes when the girdle band wall is in resonance. This mode is unlikely to manifest with conventional MEMS resonators due to fabrication difficulty.

What is more interesting is the tilt mode of the girdle band wall illustrated in Figure 6. This mode is unlikely to manifest in conventional MEMS resonators due to the 2D nature of most MEMS topology, constrained by microfabrication complexities. Therefore, this set of resonant modes is potentially a unique advantage of diatom frustules, which can open up a new avenue of dynamical exploitation. The two tilt modes in Figure 6, 25.2 MHz and 26.9 MHz, are degenerate modes of the same eigenvalue due to a slight difference in the geometry of the top valve and bottom valve. This degeneracy will happen anyway in FEA, due to the unavoidable mesh asymmetry.

It can also be noted that the tilt mode shows substantial deformation on the valves, suggesting the potential for cross-modal coupling between tilt and transverse directions. The first tilt mode is also only about five times the frequency of the fundamental mode, making it relatively feasible and accessible to excite. The 1 μm wall thickness assumed here is already on the higher range. The frequency of this tilt mode would be substantially lower for thinner girdle band wall thicknesses.

### 3.2. MATLAB Numerical Modelling of Modal Coupling

**Figure 7:**
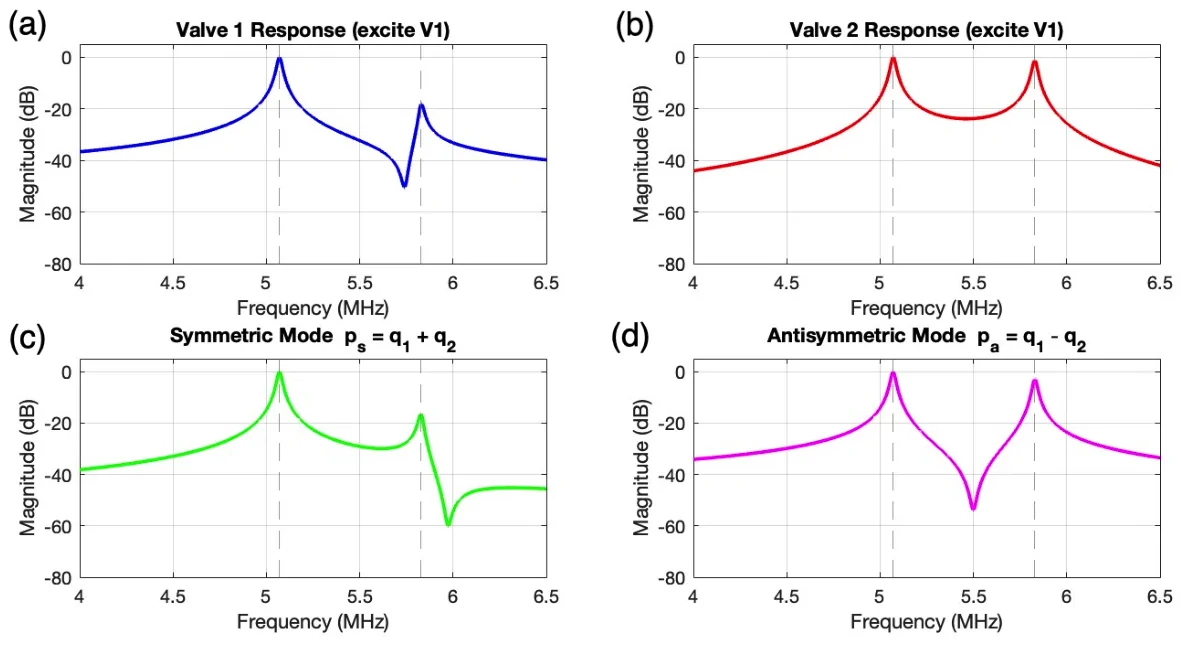
FEA-fitted MATLAB frequency response of the diatom frustule dual-membrane resonator across the fundamental valve mode range. (**a**) Single-valve excitation of valve 1, showing a primary resonance at 4.9 MHz and a secondary peak arising from inter-valve energy transfer through the girdle band coupling stiffness $k_{c}$. (**b**) Corresponding response of valve 2, showing the reciprocal coupling behaviour. (**c**) Symmetric modal coordinate $p_{s}=q_{1}+q_{2}$, isolating the lower frequency in-phase mode at 4.9 MHz. (**d**) Antisymmetric modal coordinate $p_{a}=q_{1}-q_{2}$, isolating the upper frequency out-of-phase mode at 5.5 MHz.

**Figure 8:**
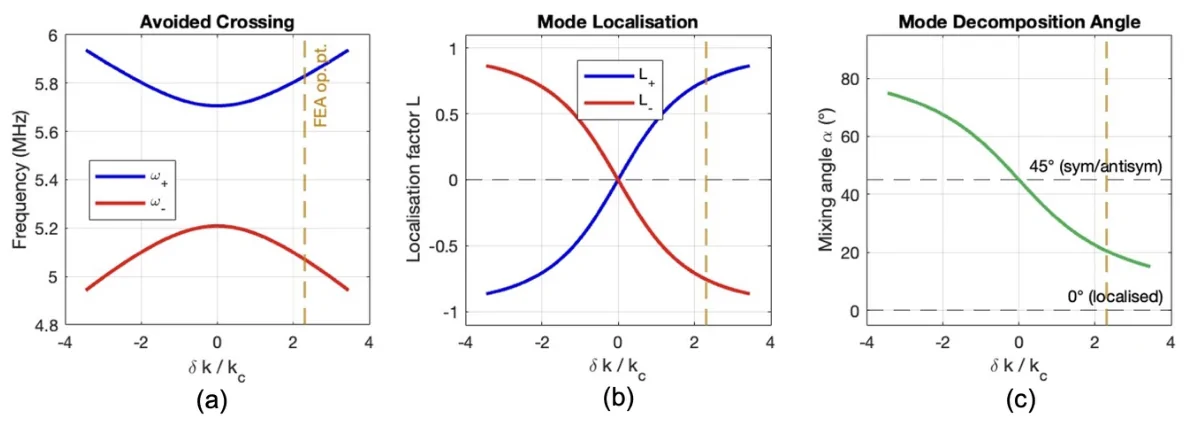
MATLAB modal analysis of the diatom frustule dual-membrane resonator as a function of normalised stiffness mismatch $\delta k/k_{c}$. (**a**) Avoided crossing of the first two eigenfrequencies, with the minimum frequency separation at δk=0 set by girdle band coupling stiffness $k_{c}$. (**b**) Mode localisation parameter $L_{\pm }$ as a function of $\delta k/k_{c}$, illustrating the transition from delocalised partial localisation (equally shared energy) to full confinement in a single valve. (**c**) Mixing angle α as a function of $\delta k/k_{c}$, transitioning from $\alpha =45^{\circ }$ (ideal symmetric/antisymmetric modes) to $\alpha =0^{\circ }$ (fully localised modes). When the frustule operates at $\alpha =20.5^{\circ }$, it sits in the maximally sensitive transition zone where mode shape change per unit stiffness perturbation is at its greatest.

Numerical values from FEA model were used to fit a MATLAB (2023b) numerical model based on the analytical equations established in the previous section. In particular, the fundamental (0, 1) modes of the valves and the tilt modes of the girdle band were closely studied to understand their 3D coupled dynamical behaviour. Figure 7 and Figure 8 show the FEA-fitted MATLAB numerical simulation of the fundamental modes of the diatom frustule dual-membrane resonator. The clean modal separations confirm well-defined normal modes consistent with a properly coupled dual-oscillator system.

Under single-valve excitation of valve 1, valve 2 produces a clear secondary peak in its frequency response. This is evidence of mechanical energy transfer through the girdle band coupling spring $k_{c}$. The relative height of this secondary peak compared to the primary peak is a direct measure of the coupling strength. This suggests that it is potentially experimentally measurable from a simple single-point drive-and-detect experiment without needing to instrument both valves simultaneously.

The symmetric and antisymmetric modal coordinates in Figure 7c,d cleanly separate the two modes. This clean separation confirms the system is behaving as a proper coupled oscillator with well-defined normal modes, and potentially provides a practical experimental strategy to measure and independently isolate each mode without modal fitting or post-processing.

The two observed modes are not entirely symmetric or antisymmetric in the ideal sense, but are instead partially localised to individual valves at 4.9 MHz and 5.5 MHz respectively. Furthermore, the avoided crossing gap can potentially be quantified by experimentally sweeping valve stiffness mismatch, suitable to serve as a research platform for resonant sensors with ultra-high sensitivity.

Looking at mode decomposition, the mixing angle sits in the maximally sensitive transition zone where small changes in either δk or $k_{c}$ that might be caused by mass or stiffness perturbations, can produce the largest possible shift in both mode frequency and mode shape simultaneously. This dual sensitivity to frequency and mode shape change has the potential to make frustules superior sensing resonators compared to single resonators, where only frequency shifts are observable.

**Figure 9:**
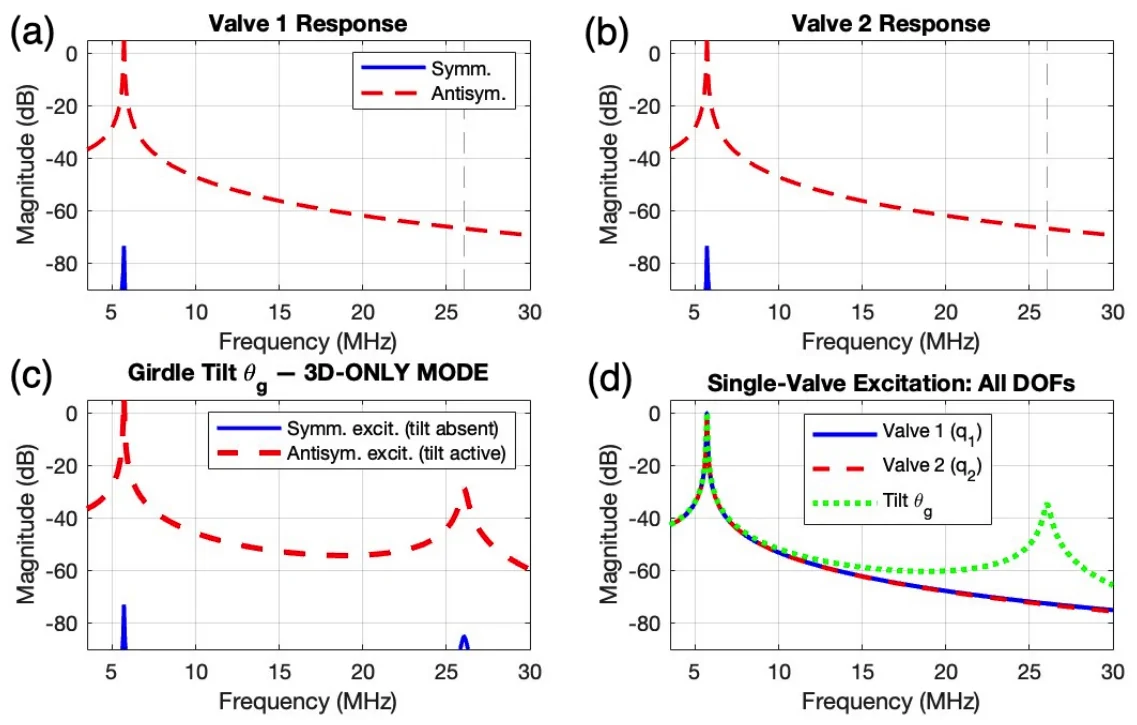
MATLAB modelling of frequency response (3.5–30 MHz) under symmetric and antisymmetric excitation for each component individually: (**a**) in valve 1, (**b**) in valve 2, (**c**) in girdle band, and (**d**) in all four DOFs together. Tilt mode is a 3D-only mode (absent in 2D MEMS resonators) and can be activated by antisymmetric excitation.

**Figure 10:**
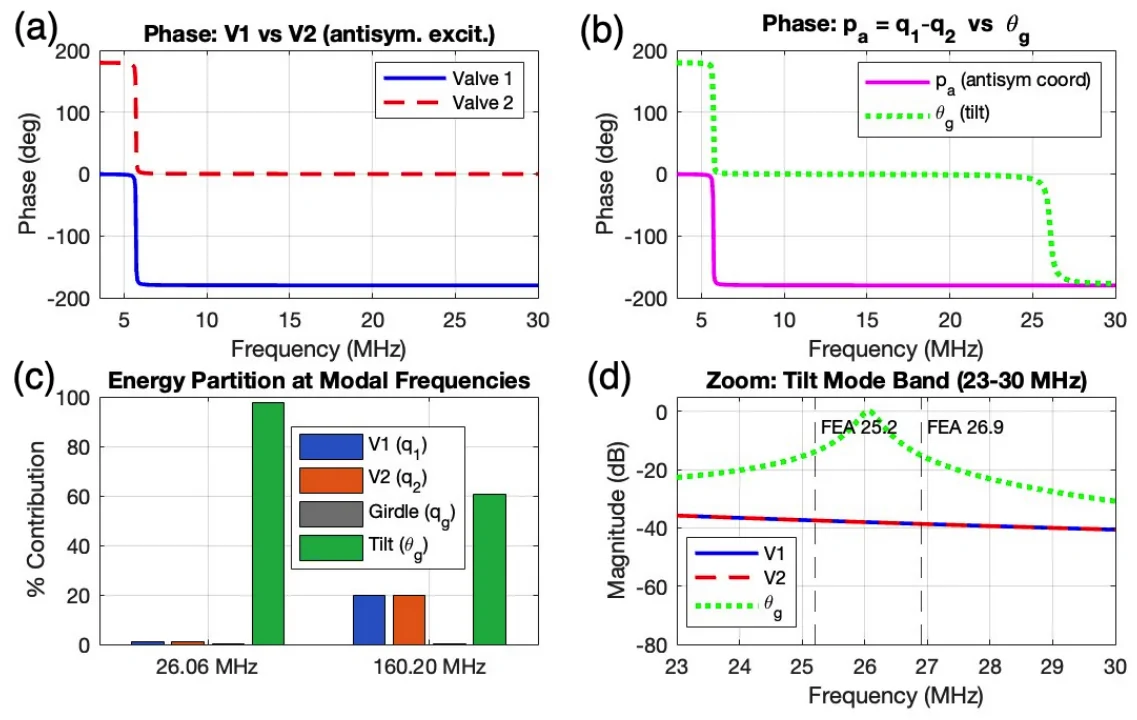
MATLAB modelling of tilt mode physics under antisymmetric excitation, exploring phase lock in (**a**,**b**), energy transfer in (**c**), and FEA validation in (**d**).

Figure 9 and Figure 10 show the FEA-fitted MATLAB numerical simulation, considering the tilt direction response from the girdle band. It can be noted that under symmetric excitation (both valves pushed equally), the tilt response is completely zero by symmetry as the girdle band has no reason to tilt when both sides are loaded equally. Under antisymmetric excitation (valves pushed in opposition), the tilt mode at 26 MHz activates fully. This selective activation by mode symmetry has no analogue in any 2D MEMS resonator because in a planar structure there is no out-of-plane axis available for this tilt to occur along. This is a direct consequence of the through-thickness 3D architecture that can be found in diatom frustule geometry. In a future potential experimental embodiment, careful selection of excitation frequency is needed, while orientation is less critical due to abundance of 3D leakage of excitation in practical MEMS devices.

The phase locking plots in Figure 10a,b illustrate that the tilt modes are true resonances and not geometric artefacts. Therefore, the girdle band participates dynamically and not simply kinematically. The energy partition plot (Figure 10c) reveals a fundamental transition in the character of mechanical energy storage as the system passes through the tilt resonance. Comparison with FEA is shown in Figure 10d, with 26 MHz as the theoretical eigenfrequeny of this mode, in contrast to the two degenerate values from the FEA plotted in dotted lines. Experimentally, degenerate modes are more likely to manifest than a single mode, due to geometric asymmetry. However, in essence, they are the same resonant mode.

At frequencies well below the tilt mode, the total mechanical energy of the system is distributed across all four degrees of freedom. The two valve displacements ($q_{1}$ and $q_{2}$) and the girdle band axial coordinate ($q_{g}$) each carry a non-negligible share, reflecting the broadly coupled, hybridised nature of the off-resonance response. This distributed energy state is the hallmark of a weakly coupled subsystem being driven away from its natural frequency, where none of the individual oscillators dominates the system’s dynamic character.

As the excitation frequency approaches 26 MHz, the energy partition undergoes a sharp and decisive redistribution. The girdle band tilt coordinate $\theta_{g}$ absorbs an increasingly dominant fraction of the total energy, while the valve and axial girdle contributions diminish correspondingly. At the peak of the tilt resonance, virtually the entire mechanical energy of the system is concentrated in $\theta_{g}$. The system behaves, to a close approximation, as a single-degree-of-freedom tilt oscillator. This energy concentration is a direct consequence of the low rotational inertia $I_{g}$ of the girdle band: a small driving moment transmitted antisymmetrically from the valves is sufficient to drive the girdle into large-amplitude tilt oscillation, producing a mechanical amplification effect entirely analogous to the mode-localised energy concentration exploited in high-sensitivity MEMS sensors.

This energy collapse onto a single coordinate has two important physical implications. First, it quantitatively justifies treating the tilt mode as a genuinely independent degree of freedom in the lumped-parameter model, rather than as a perturbation or secondary effect of the valve bending modes. The energy partition confirms it is dynamically decoupled at resonance. Second, it identifies the girdle band tilt coordinate as the optimal sensing port for any transducer seeking to exploit the tilt resonance. Instrumentation placed to measure $\theta_{g}$ directly, for example through differential capacitance across the girdle band circumference, or through a differential optical lever, will capture nearly the full mechanical energy of the mode, maximising transduction efficiency and signal-to-noise ratio. This is in direct contrast to the valve bending modes, where energy is shared between multiple coordinates and no single measurement port captures the complete modal response.

Figure 10d presents a direct validation of the lumped-parameter tilt model against the FEA eigenfrequency solution. The tilt resonance frequency predicted by the analytical model at 26 MHz closely agrees with the FEA result, confirming that the four-DOF lumped-parameter formulation, with the back-calculated rotational stiffness $k_{\phi }$, faithfully captures the essential tilt dynamics of the girdle band without requiring the full spatial complexity of the finite element solution. Any small residual discrepancy can be attributed to the Galerkin reduction assuming a single dominant mode shape, whereas the FEA captures higher-order spatial contributions to the tilt deformation.

## 4. Discussion: Towards Biogenic MEMS

Prior studies on the mechanics of diatom frustules have mainly focused on probing the material properties such as Young’s modulus, stiffness and density [25]; static behaviour such as fracture resistance and elastic deformation [24]; modal analysis [20,22]; and aggregate ensemble dynamics [21]. Dynamic analysis primarily assumed a single resonator model [20], behaving essentially the same as a standard circular plate resonator. However, this does not fully capture the rich dynamics of the 3D hollowed frustule structure.

This paper analysed the frustule as two disc membranes, vertically coupled by the walls of girdle bands. For the particular embodiment here, this revealed interesting girdle band tilt modes at 25 MHz to 27 MHz, which is a qualitatively distinct dynamic phenomenon with no counterparts in conventional planar MEMS resonators. A first analytical model is also set up to better understand the numerical behaviour of this 3D coupled system.

The present results demonstrate that the girdle band, biologically evolved primarily as a reproductive expansion joint [16], can function as a torsional coupling element whose rotational stiffness governs an entirely separate family of high-frequency modes. Crucially, the back-calculated rotational stiffness exceeds thin-wall geometric estimates, consistent with AFM and nanoindentation studies [24] that report significant nanomechanical heterogeneity across different frustule layers and attribute enhanced localised stiffness to the interlocking silica microstructure at inter-band contact regions. This finding reframes the girdle band from a passive structural connector into an active dynamic participant, making it a structurally programmable degree of freedom that has no analogue in lithographically defined MEMS architectures.

The 3D modal coupling demonstrated here places the diatom frustule within the broader class of weakly coupled resonator systems [26] that have attracted significant interest in MEMS sensing for their ability to exploit mode localisation and other coupling dynamics as a transduction mechanism.

Mode-localised MEMS sensors [27] achieve sensitivity improvements of several orders of magnitude over single-resonator frequency-shift devices [28], yet their fabrication requires precisely engineered coupling beams, symmetric resonator pairs, and carefully controlled stiffness perturbation, all achieved through expensive multi-step lithographic and etching processes. The frustule replicates this architecture biologically: two valves of near-identical geometry, vertically coupled through the girdle band spring, sitting naturally in the partial localisation regime where sensitivity to symmetry-breaking perturbations is maximised.

Each individual frustule would geometrically differ from each other, even within similarly shaped groups, making reproducibility a challenge. However, lithography and etching tolerances would also result in geometric variations amongst MEMS devices. MEMS devices definitely have superior reproducibility by relative comparison; however, when ultra-sensitivity and precision are involved, both biogenic and MEMS fabricated resonators equally demand careful calibration—irrespective of whether geometric variance is either 10 % or 0.1 %.

For the embodiment explored here, the mixing angle of 20.5° places the system neatly in the transition zone between the symmetric and fully localised limits; this is precisely the operating point at which mode shape change per unit stiffness perturbation is largest, and the point that mode-localised MEMS sensor designers deliberately target. This demonstrates that a carefully selected frustule has the potential to occupy this dynamically optimal regime, arising not from deliberate engineering design, but from nature’s evolutionary refinement. Therefore, it provides a compelling theoretical basis for its further research and development as a bio-derived coupled resonator platform.

**Figure 11:**
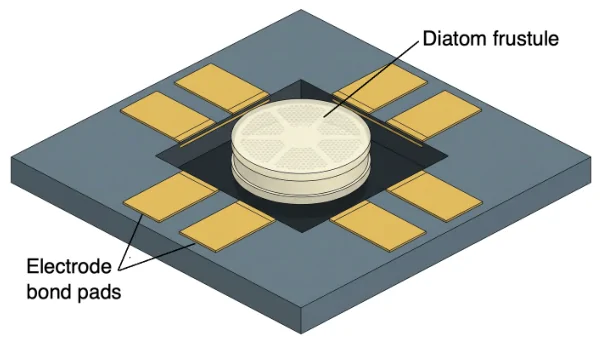
Concept diagram of the proposed hybrid biogenic MEMS device.

Through selecting a desired diatom frustule, a biogenic 3D resonator can then be assembled into a MEMS device, illustrated by the concept diagram outlined in Figure 11. Etched device substrate can be adopted to enable a suspended structure to fix the frustule along its circumferential centre line in the same manner as the modelling carried out in this paper. Functional transduction layers, such as piezoelectric material, can be added to interrogate signals from the structure. Alternatively, purely non-contact methods, such as acoustic and optical transducers, can be employed as the drive and sense ports.

This biogenic MEMS concept then shifts the burden away from microfabrication of a complex 3D structure, towards the cleaning, selection, assembly and operation of a bio-derived component. It will bring about its own challenges in terms of potential contamination from residual water and organic matter, mechanical and electrical integration challenges with current MEMS processes, and long-term resonator stability of the biosilica structure. However, it also holds the promise of a new world of untapped 3D microdynamic potentials waiting to be explored by the wider microsystem research community.

## 5. Conclusions

This work constructed, for the first time, an analytical model based on a vertically coupled dual-membrane resonator to represent the dynamic behaviour of a circular diatom frustule. As a 3D coupled resonator, nature’s microfabricated frustule is established as a dynamically rich biogenic silica (SiO_2_) structure. Its valve bending modes and partial mode localisation behaviour are analogous to engineered MEMS sensing architectures. Crucially, it also exhibits unique girdle band tilt modes that are prohibitively complex to achieve with current MEMS fabrication technologies due to an interior 3D etched volume enclosed on all sides, opening up new modal and coupling phenomena that can be exploited as transduction mechanisms. Subsequent work could involve experimental modal studies such as using microscopic laser Doppler vibrometry to investigate girdle tilt modes, alongside further nonlinear dynamic analysis of other higher modes and detailed parametric studies. Devising a method to select and assemble frustules into MEMS processes can also open up a new avenue for biogenic MEMS, bridging the gap between nature’s microstructures and humanity’s microsystems.

## Figures and Tables

**Table 1:** Parameters used in numerical modelling.

Frustule diameter (μm)	40	Young’s modulus (GPa)	73.1
Frustule height (μm)	10	Poisson’s ratio (1)	0.17
Girdle band height (μm)	8	Rotational inertia ($kg\;m^{2}$)	2.81 × 10 ^−23^
Valve thickness (μm)	1	Coupling stiffness $k_{1}$ (N m^−1^)	266.1
Hole diameter (μm)	0.8	Coupling stiffness $k_{2}$ (N m^−1^)	335.3
Density (Kg m^−3^)	2203	Coupling stiffness $k_{c}$ (N m^−1^)	30.1
Valve 1 mass $m_{1}$ (kg)	2.81 × 10 ^−13^	Axial coupling $k_{z}$ (N m^−1^)	3.25 × 10 ^5^
Valve 2 mass $m_{2}$ (kg)	2.81 × 10 ^−13^	Quality factor valve $Q_{v}$ (1)	200
Girdle mass $m_{g}$ (kg)	8.99 × 10 ^−14^	Quality factor girdle $Q_{g}$ (1)	80

## Data Availability

The raw data supporting the conclusions of this article will be made available by the authors on request.
